# Connecting METTL3 and intratumoural CD33^+^ MDSCs in predicting clinical outcome in cervical cancer

**DOI:** 10.1186/s12967-020-02553-z

**Published:** 2020-10-15

**Authors:** Huan-he Ni, Lin Zhang, He Huang, Shu-qin Dai, Jiang Li

**Affiliations:** 1 State Key Laboratory of Oncology in South China, Collaborative Innovation Center for Cancer Medicine, Department of Biotherapy, Sun Yat-Sen University Cancer Center, 651 Dongfeng East Road, Guangzhou, 510060 P. R. China; 2 Department of Biotherapy, Sun Yat-Sen University Cancer Center, 651 Dongfeng East Road, Guangzhou, 510060 China; 3 Department of Clinical Laboratory, Sun Yat-Sen University Cancer Center, State Key Laboratory of Oncology in South China, Collaborative Innovation Center for Cancer Medicine, Guangzhou, Guangdong 510060 P.R. China; 4 Department of Gynecological Oncology, Sun Yat-Sen University Cancer Center, 651 Dongfeng East Road, Guangzhou, 510060 China

**Keywords:** METTL3, CD33, Tumour microenvironment, Cervical cancer, Prognosis

## Abstract

**Background:**

Methyltransferase-like 3 (METTL3) is a member of the m^6^A methyltransferase family and acts as an oncogene in cancers. Recent studies suggest that host innate immunity is regulated by the enzymes controlling m^6^A epitranscriptomic changes. Here, we aim to explore the associations between the levels of METTL3 and CD33^+^ myeloid-derived suppressor cells (MDSCs) in tumour tissues and the survival of patients with cervical cancer (CC).

**Methods:**

Specimens of paraffin embedded tumour from 197 CC patients were collected. The expression levels of METTL3 and CD33 were measured by immunohistochemical (IHC) staining. The clinical associations of the IHC variants were analysed by Pearson’s or Spearman’s chi-square tests. Overall survival (OS) and disease-free survival (DFS) were estimated by the Kaplan–Meier method and log-rank test. Hazard ratios (HRs) and independent significance were obtained via Cox proportional hazards models for multivariate analyses. METTL3 in CD33^+^ cells or CC-derived cells was knocked down by METTL3-specific siRNA, and MDSC induction in vitro was performed in a co-culture system in the presence of METTL3-siRNA and METTL3-knockdown-CC-derived cells compared with that of the corresponding controls.

**Results:**

We found that tumour tissues displayed increased levels of METTL3 and CD33^+^ MDSCs compared with tumour-adjacent tissues from the same CC patients. Importantly, METTL3 expression was positively related to the density of CD33^+^ cells in tumour tissues (*P* = 0.011). We further found that the direct CD33^+^CD11b^+^HLA-DR^−^ MDSC induction and tumour-derived MDSC induction in vitro were decreased in the absence of METTL3. The level of METTL3 in tumour microenvironments was significantly related to advanced tumour stage. The levels of METTL3 and CD33^+^ MDSCs in tumour tissues were notably associated with reduced DFS or OS. Cox model analysis revealed that the level of METTL3 in tumour cells was an independent factor for patient survival, specifically for DFS (HR = 3.157, *P* = 0.022) and OS (HR = 3.271, *P* = 0.012), while the CD33^+^ MDSC number was an independent predictor for DFS (HR: 3.958, *P* = 0.031). Interestingly, in patients with advanced-disease stages (II–IV), METTL3 in tumour cells was an independent factor for DFS (HR = 6.725, *P* = 0.010) and OS (HR = 5.140, *P* = 0.021), while CD33^+^ MDSC density was an independent factor for OS (HR = 8.802, *P* = 0.037).

**Conclusion:**

Our findings suggest that CD33^+^ MDSC expansion is linked to high levels of METTL3 and that METTL3 and CD33^+^ MDSCs are independent prognostic factors in CC.

## Background

Cervical cancer (CC) is one of the most common tumours, ranking fourth for both incidence and mortality in women worldwide [1–3]. CC is the result of continuous infection with some strains of human papillomavirus (HPV), such as HPV16 and HPV18 [4, 5]. Though there are abundant measures of prevention and cure, cervical cancer continues to exhibit high invasion and poor prognosis [6]. In the past decade, researchers worldwide have found that the expression levels of molecular markers in the tumour microenvironment could be an essential factor for cervical cancer (CC) growth and metastasis [7, 8]. In addition to traditional prognostic factors, including age, WHO grade, TNM stage and clinical status, some of the molecular markers could be new predictors of CC prognosis [6, 9, 10]. However, there are no confirmed molecular markers for tumour progression or prognosis in CC patients. The related viral proteins E6 and E7 have been the focal points of research for the past several years [11, 12]. In other words, easily detectable and meaningful molecular markers need to be confirmed.

Methyltransferase-like 3 (METTL3) is associated with N^6^-methyladenosine (m^6^A) RNA methylation, which is the most abundant modification ubiquitously occurring in eukaryotic mRNAs [13, 14]. This modification regulates mRNA stability or translation and can affect many functions, such as immune cell differentiation, cell development, circadian periods and tumour growth [15, 16]. In previous studies, METTL3 was found to have an adverse influence on acute myeloblastic leukaemia (AML), breast cancer (BC), ovarian carcinoma, bladder cancer (BC) and gastric cancer (GC) [17–22]. Additionally, m^6^A modifications are carried out by a combination of m^6^A methyltransferases (also named writers: METTL3, METTL14 and WTAP), m^6^A demethylases (also named erasers: FTO and ALKBH5) and specific RNA-binding proteins (also named readers: YTHDF1/2/3, HNRNPA2B1, IGF2BP1/2/3, eIF3 [22–24].

CD33-positive cells are usually defined as myeloid-derived suppressor cells (MDSCs) with suppressive influence on human tumour tissues [25, 26]. MDSCs in the tumour environment were confirmed to be an independent indicator of poor prognosis in patients with many solid tumours [25, 27, 28]. In our previous studies, the MDSC proportion was expanded in the tumour microenvironment and showed extensive negative regulatory function for antitumour immunity in malignancies [29–31]. Recent studies have indicated that the differentiation of myeloid cells is regulated by m^6^A methyltransferases, including METTL3 [22, 32, 33]. We hypothesized that MDSC expansion may be linked to the level of METTL3 in the microenvironment of CC.

In the present study, we detected the levels of METTL3 and CD33^+^ MDSCs in tumour specimens from 197 CC patients by immunohistochemical (IHC) staining. We observed increased levels of METTL3 and CD33^+^ MDSCs in tumour tissues and positive associations between the levels of METTL3 and CD33^+^ MDSCs. The high levels of METTL3 and CD33^+^ MDSCs in the CC tumour microenvironment were significantly associated with poor disease-free survival (DFS) and overall survival (OS) in CC patients. Importantly, METTL3 and CD33^+^ MDSCs were independent prognostic predictors for CC patients. These findings suggest that METTL3 and MDSCs contribute to the development of disease and that METTL3 may respond to MDSC expansion in tumour microenvironments in CC.

## Methods

### Patients and tissue samples

A total of one hundred ninety-seven CC patients who received therapy at Sun Yat-Sen University Cancer Center in Guangzhou, China, and who accepted medical follow-up that continued until 2019 were included. Paraffin tumour specimens from 197 CC patients were collected at Sun Yat-Sen University Cancer Center between 2008 and 2010. In this retrospective study, none of the patients received antitumour treatment before tumour tissue was obtained, and all 197 patients were histologically confirmed as having primary CC. As shown in Table 1, according to the WHO and the International Federation of Gynecology and Obstetrics classification criteria, 127 (64.5%) patients had stage I disease, and 70 (35.5%) had stage II–IV disease; 26 (13.2%) died. Forty-five (22.8%) patients received surgery alone, 41 (20.8%) patients received radiation therapy alone, 27 (13.7%) patients received surgery + radiation therapy, and 84 (42.6%) patients received surgery + radiation therapy + chemotherapy. The tumour specimens and clinical information were provided by the Pathology Department of Sun Yat-Sen University Cancer Center. The study was approved by the Research Ethics Committee of the Sun Yat-sen University Cancer Center, and written informed consent was obtained from all 197 patients.

**Table 1:** Clinical characteristics of total 197 patients with cervical cancer

Characteristics	No. of patients (%)
Total case	197
Age (years)	
Median, range	44, 28–79
≤ 44	106 (53.8%)
> 44	91 (46.2%)
Gender	
Male	0 (0%)
Female	197 (100.0%)
Pathological tumor (T) status^a^	
T1	147 (74.6%)
T2–4	50 (25.4%)
Pathological node (N) status^a^	
N0	173 (87.8%)
N1	24 (12.2%)
Pathological metastasis (M) status^a^	
M0	197 (100.0%)
M1	0 (0%)
Clinical stage^b^	
I	127 (64.5%)
II–IV	70 (35.5%)
Death	
No	171 (86.8%)
Yes	26 (13.2%)
Therapy after surgery	
Surgery alone	45 (22.8%)
Radiation therapy alone	41 (20.8%)
Surgery & radiation therapy	27 (13.7%)
Surgery& radiation therapy & chemotherapy	84 (42.6%)

^a^Pathological Tumor (T) Status, Pathological Node (N) Status and Pathological metastasis (M) Status are from International Union against Cancer 2002 TNM staging system

^b^Clinical stage is according to International Federation of Gynecology and Obstetrics (FIGO)

The HeLa cervical cancer cell line is maintained in our laboratory. HeLa cells were cultured RPMI 1640 (Gibco, Grand Island, NY, USA) containing 10% foetal bovine serum (FBS; EXCELL BIO, Florida, USA).

### Antibodies and reagents

The antibodies and reagents used in this study were as follows. For immunoblotting, immunohistochemistry (IHC) and immunofluorescence staining, rabbit anti-METTL3 antibody (ab195352; Abcam, Cambridge, UK), rabbit anti-CD33 antibody (ab199432; Abcam, Cambridge, UK), rabbit mAb IgG control (ab172730; Abcam, Cambridge, UK), and rabbit anti-GAPDH antibody (10494; Proteintech, Wuhan, China)) were used. Rabbit anti-human IgG (H + L) secondary antibody, HRP (PV-6001-6.0, ZSGB Bio, Beijing, China), a DAB Horseradish Peroxidase Colour Development Kit (ZLI-9017, ZSGB Bio, Beijing, China), and RIPA cell lysis buffer (CW2333S CWBIO, Beijing, China) were also applied. Milk (1706404, Bio-Rad, Hercules, USA) and West dura extended duration substrate (34075, Thermo Scientific, Carlsbad, USA), haematoxylin (DH0001, Solarbio, Beijing, China) and DAPI (GB1012, Servicebio, Wuhan, China); Lipofectamine™ LTX Reagent with PLUS™ Reagent (15338100, Invitrogen, Carlsbad, USA) were used for the siRNA assay. For CD33^+^ cell isolation, human CD33-antibody-linked magnetic beads (23227, Invitrogen, Carlsbad, USA) were applied. Antibodies for flow cytometry were as follows: PerCP-Cyanine5.5 anti-human CD33 antibody (45-0338-42, eBioscience, San Diego, USA), APC anti-human HLA-DR antibody (17-9956-42, eBioscience, San Diego, USA), and PE anti-human CD11b antibody (12-0118-42, eBioscience, San Diego, USA). Fixable Viability Stain 700 (564997, BD, San Jose, USA) was used to distinguish the dead cells.

### Immunohistochemistry (IHC) and immunofluorescence staining

Paraffin-embedded tissues were continuously sectioned at a thickness of 4 μm, and the immunohistochemistry kit was used according to the manufacturer’s instructions. In brief, tissue sections were deparaffinized by xylene, rehydrated in graded alcohols and immersed in EDTA (PH 8.0). Microwave (95 °C 12 min) was applied for antigen retrieval, and samples were cooled to room temperature. The endogenous enzyme block reagent was used to block the activities of endogenous peroxidase. The goat serum was applied to block nonspecific binding sites at room temperature for 30 min. Primary antibodies, including rabbit anti-METTL3 antibody (1:400), rabbit anti-CD33 antibody (1:200), and rabbit mAb IgG control (1:200) were incubated at 37 °C for 1 h and developed with peroxidase. After staining by haematoxylin, images were taken under a microscope (NIKON ECLIPSE 80i). The expression of METTL3 on CD33^+^ cells was measured by immunofluorescence staining; DAPI was used to stain the nuclei. The images were taken with a fluorescence microscope (NIKON ECLIPSE C1).

The METTL3 expression level was scored in tumour cells in five to ten separate × 400 high-power fields (HPFs). We scored METTL3 expression in the tumour cells of each specimen using a semiquantitative immunoreactivity scoring system, which ranged from 0 to 12 and was equal to multiplication of the intensity of immunohistochemical staining (zero: no staining; one: weak staining; two: moderate staining; and three: strong staining) and the percentage of positive tumour cells (one: less than 25%; two: 25–50%; three: 50–75%; and four: more than 75%). The expression of CD33 was determined by counting CD33-positive cells from five to ten separate × 400 HPFs from the same patient. METTL3 expression level in tumour-infiltrating cells (TILs) was evaluated based on the mean percentage from five to ten separate × 400 HPFs from the same patient. These METTL3- and CD33-positive scores were determined separately by two pathologists. An isotype control IgG antibody was included.

### Knockdown of METTL3 by siRNA

To knock down METTL3 in HeLa cells or CD33^+^ cells, we generated METTL3-specific siRNA (siMETTL3) with the help of RiboBio; a control-siRNA vector was also generated. The siRNAs were transiently transfected into HeLa or CD33^+^ cells by Lipofectamine™ LTX Reagent with PLUS™ Reagent according to the manufacturer's instructions. After 48 h, the cells were harvested for immunoblotting and MDSC induction.

The sequences of siMETTL3 siRNAs were as follows:

siMETTL3_001 5′-CAAGTATGTTCACTATGAA-3′;

siMETTL3_002 5′-GACTGCTCTTTCCTTAATA -3′; and.

siMETTL3_003 5′-GGACTCGACTACAGTAGCT-3’.

### MDSCs induction in vitro and fluorescence-activated cell sort (FACS) staining

Peripheral blood mononuclear cells (PBMCs) were derived from the peripheral blood of healthy donors by gradient centrifugation separation. The CD33^+^ cells were sorted by human CD33-antibody-linked magnetic beads. After isolation, the 1 × 10^6 CD33^+^ cells were seeded in a 24-well plate (outer well) and co-cultured with HeLa cells (inner well, at 1:2 ratio) with or without METTL3 knockdown in a Transwell system (3421, Corning, New York, NY, USA) for 48 h. The cells were harvested for FACS staining and detected by cytometry CytExpert software (Beckman Coulter, San Jose, USA) or immunoblotting. Cells were pipetted into single-cell suspensions and incubated with corresponding fluorescence-labelled antibodies according to the manufacturer’s instructions. The flow cytometer used was a cytoFLEX (Beckman), and all data were analysed by the original analysis software provided with the flow cytometer (CytExpert). The CD33^+^CD11b^+^HLA-DR^−^ cells were defined as peripheral MDSCs in this study.

### Immunoblotting

The harvested cells were lysed with pre-cooled RIPA buffer, and the proteins were quantified by a BCA protein assay kit (23227, Invitrogen, Carlsbad, USA) and then separated using a 10% SDS-PAGE. Proteins were transferred onto polyvinylidene difluoride membrane (IPVH00010; Millipore, Massachusetts, USA). The membrane was blocked with 5% milk and incubated with the corresponding primary antibodies at 4 °C overnight. Next, the membrane was incubated with HRP-coupled secondary antibodies at room temperature and detected using a West dura extended duration substrate.

### Statistics

SPSS 19.0 software (SPSS Inc., Chicago, USA) was used to analyse all the data, and GraphPad Prism 7 software (La Jolla, USA) was used to obtain the curves. The median values were used as cut-off values to divide the patients into two groups (high level and low level). We used Pearson’s chi-square test or Spearman's chi-square test to analyse the relationships between immunohistochemical variants in different cell populations and patients’ clinical parameters. The relationships among the expression of METTL3 in tumour cells, METTL3 in tumour-infiltrating immune cells and CD33 in tumour-infiltrating immune cells were determined using Pearson's or Spearman’s correlation coefficient and linear regression analyses. Cut-off selection was based on X-tile (Version 3.6.1, New Haven, USA). Then, we evaluated prognostic factors in univariate and multivariate analyses using the Cox proportional hazards model. In our research, *P < 0.05 was considered significant. Raw data of this article have been deposited in the Research Data Deposit (RDD) (www.researchdata.org.cn) with accession number RDDB2020000943.

## Results

### The level of METTL3 is positively linked to the number of CD33^+^ MDSCs and contributes to tumour development

**Fig. 1:**
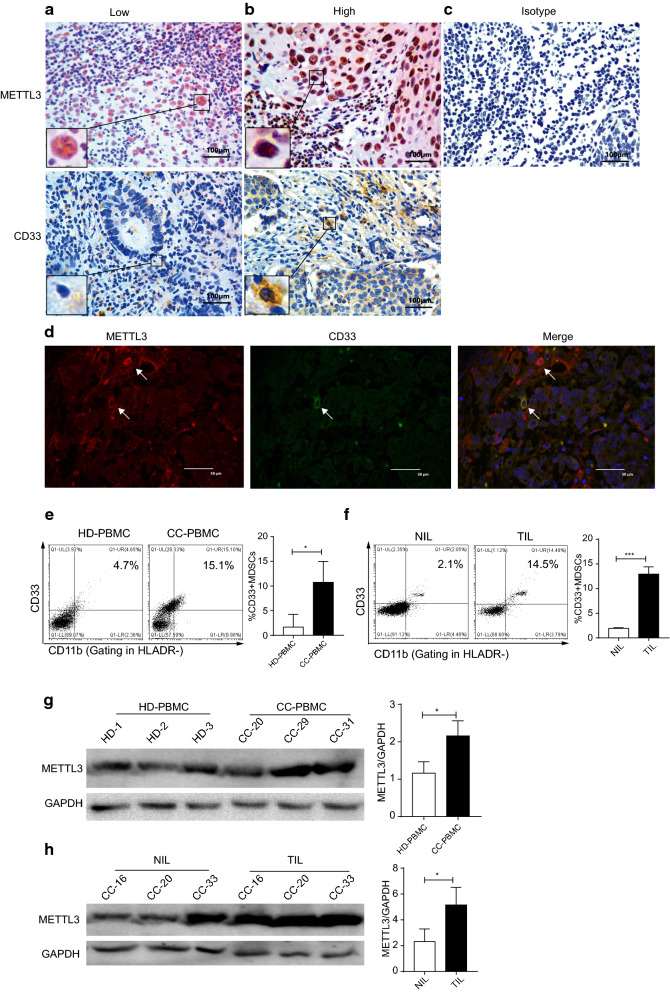
METTL3 expression and CD33^+^ MDSC distribution in patients with CC. **a**, **b** The immunohistochemical staining for METL3 and CD33 CC specimens (× 400). **c** The isotype antibody IgG was included (× 400). **d** Immunofluorescence staining for METTL3 (red) and CD33^+^ (green) in CC specimens; the white arrows point to the METTL3^+^ and CD33^+^ cells. The images were taken by fluorescence microscope. HLA-DR^−^CD33^+^CD11b^+^ cells were gated by a FACS gating strategy and were defined as MDSCs in this study. **e**, **f** Representative density plots showed the MDSC population in the peripheral blood of healthy donors (HD) or CC patients, as well as in the immune cells from tumour tissues (TIL) or tumour-adjacent tissues (NIL). A statistical graph is included for the comparison between the indicated groups. (G-H) Representative immunoblotting shows the expression of METTL3 in the peripheral blood, TILs and NILs. A statistical graph is included for the comparison between the indicated groups. The experiments in **e**, **f** were performed at least three times, and the data were plotted as the mean ± SEM. Statistics were conducted with an unpaired Student’s t test, *P < 0.05, and ***P < 0.001 vs. the corresponding control

In the present study, the levels of METTL3 and CD33^+^ MDSCs were examined in tumour specimens from 197 patients with CC by IHC. METTL3 was located in the nuclei of tumour cells and tumour-infiltrating immune cells, while CD33^+^ cells were scattered mainly in the tumour stroma (Fig. 1a, b); isotype IgG was used as a control (Fig. 1c). Importantly, we found that CD33 and METTL3 co-localized in some tumour-infiltrated immune cells (Fig. 1d). We further demonstrated that the percentage of CD33^+^CD11b^+^HLA-DR^−^ peripheral MDSCs was increased in CC patients compared with healthy donors, as was the percentage of tumour-derived CD33^+^CD11b^+^HLA-DR^−^ MDSCs compared with that of tumour-adjacent tissues (Fig. 1e, f, n = 3). Consistent with the increase in the MDSC population in CC patients, the level of METTL3 was increased in the peripheral and tumour-infiltrating immune cells compared with the corresponding controls (Fig. 1g, h). Among the 197 patients with CC, the median survival time was 96 months (range: 0–120 months), and the 10-year DFS and 10-year OS rates were 88.83 and 86.80%, respectively (Additional file 1: Figure S1A and S1B). Table 2 shows the results of the relationships between clinicopathological features and immunohistochemical variants in different cell types in the tumour microenvironment. High METTL3 expression in the tumour and in tumour-infiltrating immune cells was linked to tumour stage (*P* = 0.040 and 0.020, respectively).

**Table 2:** Correlations the expression of METTL3, CD33 and clinical parameters in total 197 cervical cancer patients

Clinicopathologic parameter	Total case	High METTL3 levels in tumor (%)	*P*	High METTL3 levels in tumor-infiltrating immune cells (%)	*P*	High density of CD33^+^ MDSCs (%)	*P*
Age							
≤ 44 (years)	86	31 (43.7%)	0.999^a^	41 (47.7%)	0.524^a^	46 (53.5%)	0.672^a^
> 44 (years)	111	40 (56.3%)		58 (52.3%)		56 (50.5%)	
T1	147	59 (40.1%)	*0.040*^a^	81 (55.1%)	*0.020*^a^	78 (53.1%)	0.536^a^
T2–4	50	12 (24.0%)		18 (36.0%)		24 (48.0%)	
N status							
N0	173	64 (37.0%)	0.454^a^	88 (50.9%)	0.644^a^	88 (50.9%)	0.493^a^
N1	24	7 (29.2%)		11 (45.8%)		14 (58.3%)	
Clinical stage							
I	127	47 (37.0%)	0.703^a^	68 (53.5%)	0.214^a^	63 (49.6%)	0.412^a^
II–IV	70	24 (34.3%)		31 (44.3%)		39 (55.7%)	

^a^Data are analyzed by Pearson’s chi-squared test

^b^Data are analyzed by Spearman’s chi-squared test

**Fig. 2:**
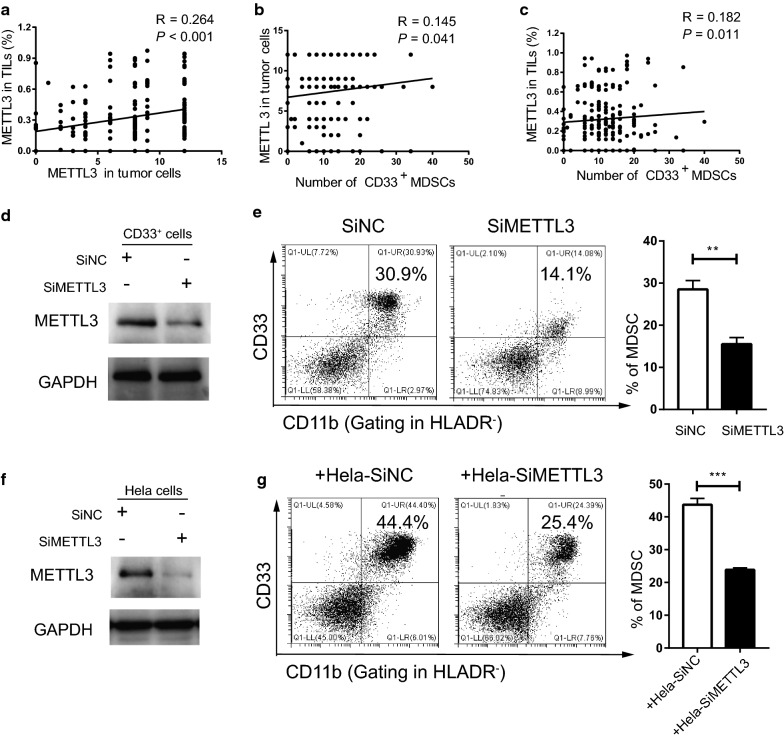
Tumour METTL3 level was positively related to intratumoural CD33^+^ MDSCs in vivo and CC-derived MDSCs in vitro. **a** The association between METTL3 expression in tumour cells and the expression of METTL3 in TILs (R = 0.264, *P* < 0.001). **b** The association between METTL3 expression in tumour cells and intratumoural CD33^+^ MDSC number (R = 0.145, *P* = 0.041). **c** The association between METTL3 expression in TILs and intratumoural CD33^+^ MDSC number (R = 0.182, *P* = 0.011). CD33^+^ cells were isolated from PBMCs of healthy donors with human anti-CD33 beads, and the METTL3 levels in CD33^+^ cells or HeLa cells were knocked down by siMETTL3. **d** Immunoblotting showed the METTL3 expression in CD33^+^ cells with or without METTL3 knockdown. **e** HLA-DR^−^CD33^+^CD11b^+^ MDSC induction from CD33^+^ cells in the presence of siMETTL3 or siControl (SiNC). A statistical graph is included for the comparison between the indicated groups. **f** Immunoblotting showed METTL3 expression in HeLa cells with or without METTL3 knockdown. **g** Tumour-associated HLA-DR^−^CD33^+^CD11b^+^ MDSC induction from CD33^+^ cells in coculture with Hela-siMETTL3 or Hela-siControl cells in a Transwell System for 48 h. A statistical graphs is included for the comparison between the indicated groups. Representative flow cytometry density plots (left) and statistical bar chart (right). The statistical analysis was performed using Spearman’s correlation and linear regression. R, Spearman’s correlation, is the correlation coefficient. The experiments in **e**, **g** were performed at least three times, and the data were plotted as the mean ± SEM. Statistics were conducted with an unpaired Student’s t test, **P < 0.01, and ***P < 0.001 vs. the corresponding control

In addition, we analysed the relationship between METTL3 expression in tumour cells and in tumour-infiltrating immune cells and the number of CD33^+^ MDSCs via Spearman’s correlation coefficient and linear regression. The expression of METTL3 in tumour cells was positively correlated with that in tumour-infiltrating immune cells (R = 0.264, *P* < 0.001) (Fig. 2a). The number of CD33^+^ cells was positively correlated with the expression of METTL3 in tumour cells (R = 0.145, *P* = 0.041) and tumour-infiltrating immune cells (R = 0.182, *P* = 0.011) (Fig. 2b, c). Moreover, we found that the METTL3 level in tumour cells was positively correlated with TILs in the early (R = 0.049, *P* = 0.012) and advanced stage (R = 0.129, *P* = 0.002) (Additional file 1: Figure S1E and S1F), while we found that in the advanced stage, the number of CD33^+^ cells was positively correlated with the METTL3 level in TILs (R = 0.088, *P* = 0.013) (Additional file 1: Figure S1J).

To further investigate the role of METTL3 in the regulation of MDSC expansion, we knocked down METL3 expression in CD33^+^ cells or HeLa cells. We found that CD33^+^CD11b^+^HLA-DR^−^ MDSCs and tumour-derived MDSCs were decreased when METTL3 was knocked down in CD33^+^ cells or HeLa cells (Fig. 2d–g).

### High METTL3 levels and CD33^+^ MDSC density are associated with poor outcomes

**Fig. 3:**
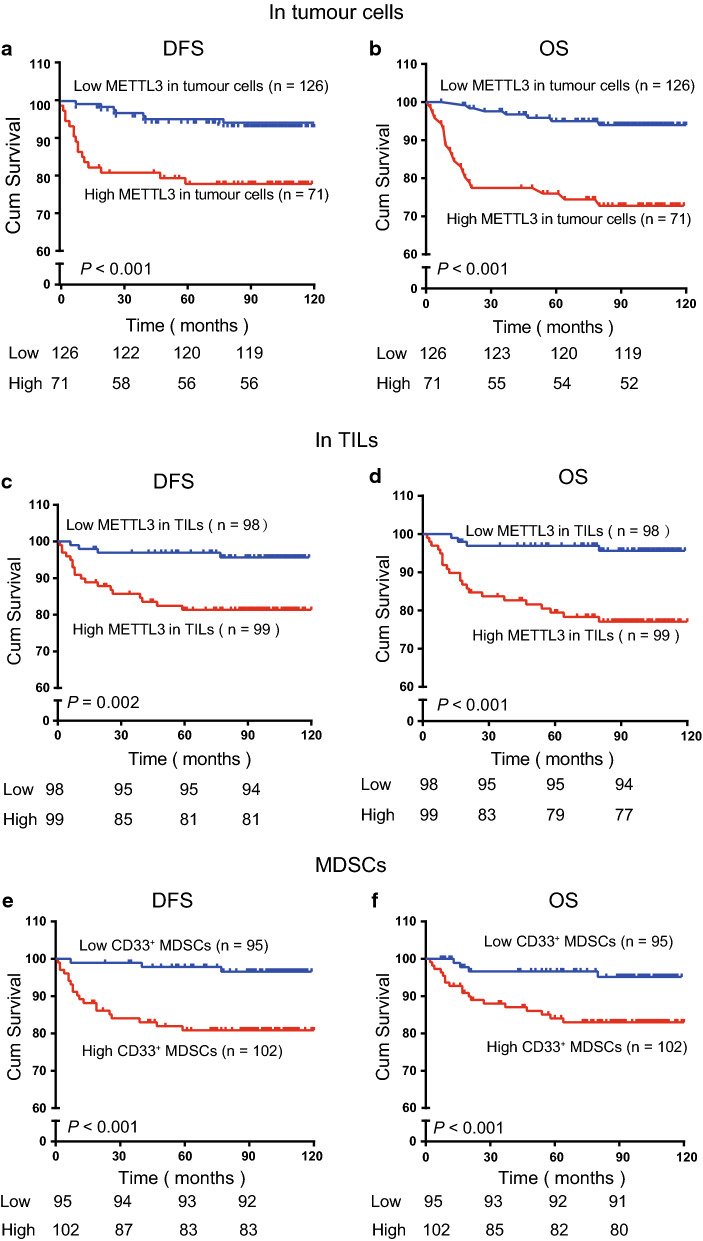
METTL3 and intratumoural CD33^+^ MDSCs were predictors for poor survival of CC patients. **a**, **b** Kaplan–Meier curves showing the relationship of DFS (*P* < 0.001, n = 197) and OS (*P* < 0.001, n = 197) of CC patients and METTL3 expression in tumour cells. **c**, **d** Kaplan–Meier curves showing the relationship of DFS (*P* = 0.002, n = 197) and OS (*P* < 0.001, n = 197) of patients and METTL3 expression in TILs. **e**, **f** Kaplan–Meier curve showing the relationship of DFS (*P* < 0.001, n = 197) and OS (*P* < 0.001, n = 197) of CC patients and intratumoural CD33^+^ MDSC density. The percentages of DFS and OS were calculated by the Kaplan–Meier method, and *P* values were calculated by the log-rank statistic. Cut-off selection was based on X-tile

To evaluate the expression of METTL3 and CD33 as predictors for the prognosis of the 197 patients, Kaplan–Meier survival curves were used for analysis. The high level of METTL3 in tumour cells was significantly correlated with decreased DFS (*P* < 0.001, Fig. 3a) and OS (*P* < 0.001, Fig. 3b) in CC patients. Accordingly, a high level of METTL3 in tumour-infiltrating immune cells was negatively correlated with DFS (*P* = 0.002, Fig. 3c) and OS (*P* < 0.001, Fig. 3d) in CC patients. The high density of CD33^+^ MDSCs was obviously correlated with decreased DFS (*P* < 0.001, Fig. 3e) and OS (*P* < 0.001, Fig. 3f) in CC patients.

### METTL3 and CD33^+^ MDSCs are independent factors for patient prognosis

As shown in Table 3, univariate analysis showed that in addition to lymph node involvement and clinical stage, high levels of METTL3 in tumour cells (HR: 4.244, *P* = 0.002) and in tumour-infiltrating immune cells (HR: 4.857, *P* = 0.004) and a high density of CD33^+^ MDSCs (HR: 6.579, *P* = 0.002) were noticeably correlated with reduced DFS. In addition, we found that high levels of METTL3 in tumour cells (HR: 5.502, *P* = 0.001) and in tumour-infiltrating immune cells (HR: 6.021, *P* = 0.001) and a high density of CD33^+^ MDSCs (HR: 5.755, *P* = 0.001) were associated with decreased OS. Moreover, clinicopathological parameters such as clinical stage (HR: 3.511, *P* = 0.005) and nodal status (HR: 2.798, *P* = 0.032) also had prognostic value with decreased DFS, and clinical stage (HR: 3.820, *P* = 0.001) was related to decreased OS. In addition, other clinical characteristics, such as age and tumour status, were not clearly related to DFS and OS (Table 3). When we performed multivariate Cox proportional hazards regression analysis in Table 4, we included all the significant univariate variables. For all 197 patients, in addition to clinical stage (HR: 3.827, *P* = 0.003), N status (HR: 3.219, *P* = 0.021) was an independent factor for DFS, clinical stage (HR: 4.248, *P* < 0.001) was an independent factor for OS, and METTL3 levels in tumour cells (HR: 3.157, *P* = 0.022) and in tumour-infiltrating immune cells (HR: 3.368, *P* = 0.036) and CD33^+^ MDSCs (HR: 3.958, *P* = 0.031) were independent factors for both DFS and OS (Table 4).

**Table 3:** The univariate cox regression analysis in cervical cancer patients

Factors	Disease-free survival		Overall survival	
	HR (95%CI)	*P* value	HR (95%CI)	*P* value
The levels of METTL3, CD33 in tumor and tumor-infiltrating immune cells (n = 197)				
Age, years (≤ 44/ > 44)	0.937 (0.405–2.169)	0.879	1.281 (0.581–2.825)	0.539
Clinical stage (I/II–IV)	3.511 (1.472–8.373)	*0.005*	3.820 (1.702–8.573)	*0.001*
Tumor (T) status (1/2–4)	0.128 (0.017–0.955)	0.051	0.106 (0.014–0.783)	0.058
Nodal (N) status (0/1)	2.798 (1.095–7.152)	*0.032*	2.225 (0.893–5.542)	0.086
METTL3 in tumor cells (low/high)	4.244 (1.730–10.413)	*0.002*	5.502 (2.312–13.092)	*0.001*
METTL3 in tumor-infiltrating immune cells (low/high)	4.857 (1.643–14.353)	*0.004*	6.021 (2.074–17.474)	*0.001*
Number of CD33^+^ MDSCs (low/high)	6.579 (1.946–22.241)	*0.002*	5.755 (1.982–16.705)	*0.001*
Combination of METTL3 levels and CD33^+^MDSCs (low/ middle/ high)	4.672(2.149–10.156)	< *0.001*	4.890(2.369–10.093)	< *0.001*
The levels of METTL3, CD33 in tumor and tumor-infiltrating immune cells in stage I (n = 127)				
Age, years (≤ 44/ > 44)	0.364 (0.074–1.807)	0.217	0.562 (0.140–2.250)	0.416
Tumor (T) status (1/2–4)	0.033 (0.00–35.599)	0.338	0.032 (0.000–22.782)	0.305
Nodal (N) status (0/1)	2.736 (0.552–13.569)	0.218	2.325 (0.483–11.20)	0.293
METTL3 in tumor cells (low/high)	1.696 (0.424–6.783)	0.455	2.184 (0.586–8.132)	0.244
METTL3 in tumor-infiltrating immune cells (low/high)	6.489 (0.799–52.832)	0.080	7.452 (0.932–59.588)	0.058
Number of CD33^+^ MDSCs (low/high)	1.732 (0.414–7.250)	0.452	2.085 (0.521–8.339)	0.299
Combination of METTL3 levels and CD33^+^MDSCs (low/ middle/ high)	2.639 (0.896–7.775)	0.078	3.071(1.056–8.931)	*0.039*
The levels of METTL3, CD33 in tumor and tumor-infiltrating immune cells in stage II–IV (n = 70)				
Age, years (≤ 44/ > 44)	0.953 (0.299–3.039)	0.935	1.240 (0.404–3.804)	0.707
Tumor (T) status (1/2–4)	0.145 (0.019–1.110)	0.063	0.116 (0.015–0.874)	0.537
Nodal (N) status (0/1)	2.554 (0.800–8.152)	0.113	1.192 (0.623–5.869)	0.257
METTL3 in tumor cells (low/high)	9.713 (2.697–34.978)	*0.001*	13.199 (3.776–46.130)	< *0.001*
METTL3 in tumor-infiltrating immune cells (low/high)	5.373 (1.498–19.276)	*0.010*	7.209 (2.070–25.106)	*0.002*
Number of CD33^+^ MDSCs (low/high)	66.197 (0.891–4917.536)	0.056	16.621 (2.199–125.660)	*0.006*
*Combination of METTL3 levels and CD33^+^MDSCs (low/ middle/ high)	7.673 (2.420–24.324)	*0.001*	7.286(2.667–19.902)	< *0.001*

HR hazard ratio, *95%CI* 95% confidence interval

*Since combined expression of METTL3 and CD33 in tumor-infiltrating immune cells is divided into three groups (low/middle/high), it is no longer calculated in multivariate analysis

**Table 4:** The multivariate cox regression analysis in cervical cancer patients

Factors	Disease-free survival		Overall survival	
	HR (95%CI)	*P* value	HR (95%CI)	*P* value
The levels of METTL3, CD33 in tumor and tumor-infiltrating immune cells (n = 197)				
Clinical stage (I/II–IV)	3.827 (1.585–9.241)	*0.003*	4.248 (1.886–9.571)	< *0.001*
Tumor (T) status (1/2–4)	–	**–**	**–**	**–**
Nodal (N) status (0/1)	3.219 (1.197–8.653)	*0.021*	**–**	**–**
METTL3 in tumor cells (low/high)	3.157 (1.181–8.438)	*0.022*	3.271 (1.303–8.213)	*0.012*
METTL3 in tumor-infiltrating immune cells (low/high)	3.368 (1.080–10.502)	*0.036*	1.006(0.202–5.013)	0.994
Number of CD33^+^ MDSCs (low/high)	3.958(1.138–13.767)	*0.031*	**–**	**–**
The levels of METTL3, CD33 in tumor and tumor-infiltrating immune cells in stage II–IV (n = 70)				
Tumor (T) status (1/2–4)	**–**	**–**	0.173 (0.023–1.317)	0.090
METTL3 in tumor cells (low/high)	6.725 (1.576–28.696)	*0.010*	5.140 (1.286–20.548)	*0.021*
METTL3 in tumor-infiltrating immune cells (low/high)	2.053 (0.483–8.726)	0.330	2.149 (0.541–8.540)	0.277
Number of CD33^+^ MDSCs (low/high)	**–**	**–**	8.802 (1.136–68.233)	*0.037*

The significant different factors in univariate analysis were analyzed by multivariate analysis, and the factors which were not significant in univariate analysis were not included

### METTL3 and CD33^+^ MDSCs have predictive value for patients with early and advanced disease stages

**Fig. 4:**
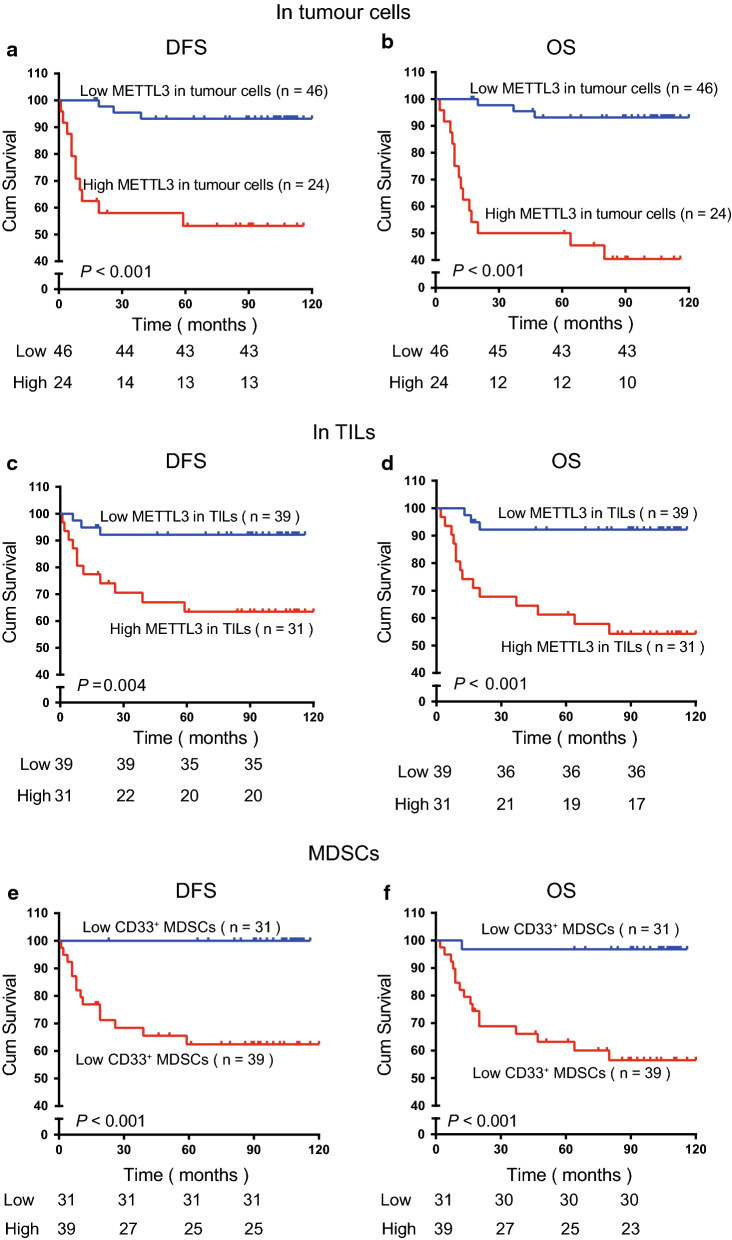
METTL3 and intratumoural CD33^+^ MDSCs were predictors for poor survival of CC patients with advanced disease stage. **a**, **b** Kaplan–Meier curves showing the relationship of DFS (*P* < 0.001, n = 70) and OS (*P* < 0.001, n = 70) of CC patients in the advanced disease stage and METTL3 expression in tumour cells. **c**, **d** Kaplan–Meier curves showing the relationship of DFS (*P* = 0.004, n = 70) and OS (*P* < 0.001, n = 70) of patients in the advanced disease stage and METTL3 expression in TILs. **e**, **f** Kaplan–Meier curves showing the relationship of DFS (*P* < 0.001, n = 70) and OS (*P* < 0.001, n = 70) of CC patients in the advanced disease stage and intratumoural CD33^+^ MDSC density. The percentages of DFS and OS were calculated by the Kaplan–Meier method, and *P* values were calculated by the log-rank statistic. Cut-off selection was based on X-tile

We further divided the 197 patients into two subgroups based on the clinicopathological stage: 127 of the total patients were in early disease stage (stage I), while 70 of the total patients were in advanced disease stage (stage II–IV). Through the Kaplan–Meier method, we found that the high expression of METTL3 in tumour-infiltrating immune cells was significantly correlated with poor DFS (*P* = 0.033) and OS (*P* = 0.019) (Additional file 2: Figure S2C and S2D) in patients with early disease stage, while there was no significant association between the high expression of METTL3 in tumour cells (*P* = 0.400 vs *P* = 0.183) and the number of CD33^+^ MDSCs (*P* = 0.393 vs *P* = 0.227) with the DFS and OS of patients with early-stage disease (Additional file 2: Figure S2A, S2B, S2E and S2F). For patients with advanced-stage disease (n = 70), a high level of METTL3 in tumour cells was dramatically correlated with decreased DFS (*P* < 0.001, Fig. 4a) and OS (*P* < 0.001, Fig. 4b), and a high level of METTL3 in tumour-infiltrating immune cells was negatively correlated with DFS (*P* = 0.004, Fig. 4c) and OS (*P* < 0.001, Fig. 4d); the increased number of CD33^+^ MDSCs was dramatically correlated with poor DFS (*P* < 0.001, Fig. 4e) and OS (*P* < 0.001, Fig. 4f). Using multivariate Cox regression analysis in the 70 patients with advanced-stage disease, METTL3 expression in tumour cells (HR: 6.725, *P* = 0.010) was an independent prognostic factor for DFS, while METTL3 expression in tumour cells (HR: 5.140, *P* = 0.021) and CD33^+^ MDSCs (HR: 8.802, *P* = 0.037) were independent prognostic factors for OS (Table 4).

### The combination of METTL3 levels and CD33^+^ MDSCs was associated with the survival of patients with CC

**Fig. 5:**
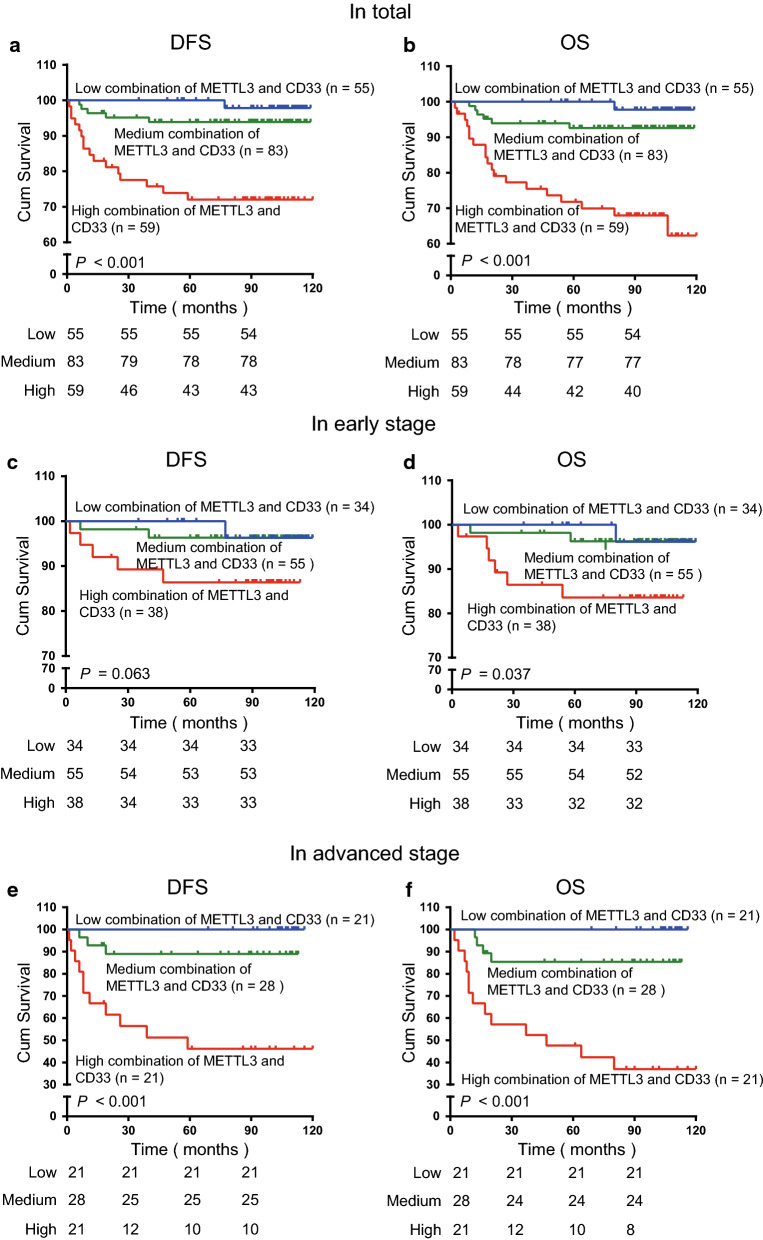
The combination of METTL3 levels and CD33^+^ MDSC numbers displayed a prognostic value for poor survival of CC patients. **a**, **b** Kaplan–Meier curves showing the relationship of DFS (*P* < 0.001, n = 197) and OS (*P* < 0.001, n = 197) of CC patients and the combination of METTL3 levels and CD33^+^ MDSC number in 197 patients. **c**, **d** Kaplan–Meier curves showing the relationship of DFS (*P* = 0.063, n = 197) and OS (*P* = 0.037, n = 197) of CC patients and the combination of METTL3 levels and CD33^+^ MDSC number in 127 early-stage patients. **e**, **f** Kaplan–Meier curves showing the relationship of DFS (*P* < 0.001, n = 197) and OS (*P* < 0.001, n = 197) of CC patients and the combination of METTL3 levels and CD33^+^ MDSC number in 70 patients with advanced-stage CC

Finally, considering that METTL3 levels were positively correlated with high CD33^+^ MDSC infiltration, we calculated the significance of the combination of these two biomarkers for the survival of CC patients. All 197 patients were divided into three groups. Patients with low levels of both METTL3 in tumour-infiltrating immune cells and CD33^+^ MDSCs were included in the combined low expression group, those with high levels of only one of the two biomarkers were included in the combined medium expression group, and those with high levels of both were included in the combined high expression group. The high combination of METTL3 and intratumoural CD33^+^ MDSCs was associated with reduced DFS (*P* < 0.001, Fig. 5a) and OS (*P* < 0.001, Fig. 5b). In the patients (127) with early-stage disease, the high combination of METTL3 and CD33^+^ MDSCs was not related to DFS (*P* = 0.063, Fig. 5c) but was clearly negatively related to OS (*P* = 0.037, Fig. 5d). In the patients (70) with advanced-stage disease, the combination of high METTL3 levels and CD33^+^ MDSCs was clearly related to unfavourable DFS (*P* < 0.001, Fig. 5e) and OS (*P* < 0.001, Fig. 5f).

Indeed, compared to METTL3 or CD33^+^ MDSCs, the combination of METTL3 and CD33^+^ MDSCs can improve the prognostic stratification of survival for CC patients, especially those in advanced disease stages. Based on a total of 197 patients, the combination of high METTL3 levels and CD33^+^ MDSCs was a predictor of worse patient prognosis, including DFS [HR (95% CI): 4.672 (2.149–10.156), *P* < 0.001] and OS [HR (95% CI): 4.890 (2.369–10.093), *P* < 0.001]. In the patients with early-stage disease, we found that the combination of high METTL3 levels and intratumoural CD33^+^ MDSCs was negatively correlated with OS [HR (95% CI): 3.071 (1.056–8.931), *P* = 0.039], but there was no significant association with DFS. Importantly, we found that the combination of high tumour METTL3 levels and intratumoural CD33^+^ MDSCs was significantly correlated with poor DFS [HR (95% CI): 7.673 (2.420–24.324), *P* = 0.001] and OS [HR (95% CI): 7.286 (2.667–19.902), *P* < 0.001] in patients with advanced disease stages (Table 3), suggesting that the combination of high METTL3 levels and CD33^+^ MDSCs improved patient prognostic stratification in those with advanced disease.

## Discussion

The development of tumour cells depends on the tumour microenvironment, which includes tumour cells, various other cells and extracellular components [7]. The immunosuppressive cells in the tumour microenvironment, such as Tregs and MDSCs, not only affect each other, but their changes in number and types will affect tumour development [34, 35]. METTL3 is one of the ‘writers’, and its role is to catalyse the m^6^A methylation of mRNA (and other nuclear RNAs); after the methylation of m^6^A, RNAs will nucleate and transport to the cytoplasm faster and then produce more proteins for function and proliferation. Some studies have shown that METTL3 expression can promote tumour cell proliferation, leading to poor patient prognosis. The tumour-infiltrated MDSC population usually induces antitumour immunity tolerance by inhibiting the proliferation and function of T cells, such as hindering antigen presentation by antigen-presenting cells [36]. Increased METTL3 levels and CD33^+^ MDSCs have been found in tumour microenvironments and lead to a poor prognosis [37–40]. In this study, we focused on the distribution of METTL3 and CD33^+^ MDSCs in the tumour microenvironment of 197 patients with CC. The positive association between METTL3 levels and CD33^+^ MDSCs and the prognostic value of these two variants in CC patients were demonstrated. Importantly, we demonstrated that knockdown of METTL3 in CD33^+^ cells or HeLa cells could attenuate MDSC or tumour-associated MDSC differentiation in vitro.

M^6^A methyltransferases, especially METTL3, can affect many physiological and pathological diseases through *p53* and other genes [14]. At the nucleic acid level, silencing m^6^A methyltransferase significantly affects gene expression and mRNA splicing patterns, leading to changes in normal cell signalling pathways and apoptosis [33]. In bladder cancer cells, m^6^A-modified direct targets IKBKB and RELA (two key regulators of the NF-κB pathway) mediated by METTL3 become factors that promote tumour development [13]; in glioblastoma stem cells (GSCs), knocking down METTL3 can induce changes in m^6^A-enriched mRNA and alter the mRNA expression of genes with key biological functions in GSCs (such as ADAM1937) [41]. In recent studies, high METTL3 levels were related to tumour invasion and poor outcomes in breast cancer and acute myeloid leukaemia (AML) [21, 42]. Our results are consistent with the results of these studies, showing that high METTL3 expression results in poor prognosis in CC patients. METTL3 regulates haematopoietic stem cell differentiation and induces the development of leukaemic cells by upregulating MYC expression [42, 43]. Therefore, we wondered whether METTL3 expression may be linked to the density of tumour-infiltrated MDSCs. Our data identified a positive association between METTL3 expression in tumour cells and in tumour-infiltrating immune cells and intratumoural CD33^+^ MDSC density. The results indicate that METTL3 could directly induce CD33^+^CD11b^ +^ HLA-DR^−^ MDSC differentiation or tumour-associated MDSC differentiation in vitro. Moreover, both METTL3 and CD33^+^ MDSCs were independent factors for the prognosis of CC patients, and the combination of METTL3 levels and CD33^+^ MDSC density displayed prognostic value for CC patients, including patients at early or late disease stages. The function, distribution and clinical relevance of the proportion of tumour-derived CD33^+^ MDSCs have been explored in recent years. MDSCs are generally elevated in tumour tissues and in the peripheral blood of cancer patients and are linked to antitumour immunity suppression, resulting in tumour growth and metastasis [25, 34, 44]. In our study, CC patients with a high infiltration of MDSCs in the cervical cancer microenvironment showed a poor prognosis, which is consistent with observations in other solid cancers. The tumour microenvironment is a main battleground between tumour cells and the host immune system. Tumour cells usually ‘educate’ infiltrated immune cells through many factors, such as cytokines or tumour-derived exosomes, to affect the proliferation, differentiation and function of tumour-infiltrating immune cells, resulting in the expansion of suppressive immune cells, including M2 macrophages, MDSCs and Tregs, and limiting the antitumour effect of cytotoxic T cells. Epigenetic modifications such as RNA modification, DNA methylation and histone modifications can rapidly regulate infiltrated immune cell differentiation and activities in tumour microenvironments [45]. Here, our data suggest that METTL3-mediated m^6^A RNA modification is positively associated with the increase in MDSC expansion and affects tumour development and prognosis in CC and induces CD33 ^+^ cells to differentiate into MDSCs in the tumour microenvironment. We further demonstrate the prognostic value of the combination of the METTL3 level in tumour-infiltrating immune cells and CD33^+^ MDSC density in CC patients, especially for those in advanced disease stages. A mechanistic study to support the role of METTL3 in the regulation of tumour-derived MDSC differentiation is currently underway, and the underlying mechanisms will be clarified in the near future.

## Conclusions

Our study demonstrated a comprehensive result of the relationship between METTL3 and CD33 in CC and revealed that METTL3 could induce direct MDSC and tumour-associated MDSC differentiation in vitro. The results showed that both biomarkers were adverse indicators for prognosis and may have significant relationships in the microenvironment of CC. Our research may offer clues for further research into the mechanism behind METTL3 in the regulation of MDSC-mediated immune suppression in the CC microenvironment.

## Supplementary information

**Additional file 1: Figure S1.** The DFS and OS curves of 197 CC patients, and expression of METTL3 and CD33 in tumours or in tumour-adjacent tissues. (A and B) Disease-free survival (DFS, A) and overall survival (OS, B) curves of 197 CC patients in this study. (C and D) Statistical analysis showing the comparison between the expression of METTL3 (*P* = 0.999, n = 18) and the number of CD33^+^ MDSCs (*P* = 0.295, n = 18) in tumour-adjacent tissues (Non-tumour) and tumour tissues (Tumour). (E and F) The association between METTL3 expression in tumour cells and the expression of METTL3 in TILs in early stage (E, R = 0.049, *P* = 0.012) or in advanced stage (F, R = 0.129, *P* = 0.002). (G and H) The association between METTL3 expression in tumour cells and intratumoural CD33^+^ MDSC number in early stage (G, R = 0.011, *P* = 0.222) or in advanced stage (H, R = 0.048, *P* = 0.070). (I and J) The association between METTL3 expression in TILs and intratumoural CD33^+^ MDSC number in early stage (I, R = 0.013, *P* = 0.195) or in advanced stage (J, R = 0.088, *P* = 0.013). Statistics were conducted with a paired Student’s t test in C and D. The correlation statistical analysis was performed using Spearman’s correlation and linear regression. R, Spearman’s correlation, is the correlation coefficient.

**Additional file 2: Figure S2.** Kaplan–Meier curves of DFS and OS according to METTL3 expression in different cell populations and intratumoural CD33^+^ cells of early-stage patients. (A and B) Kaplan–Meier curves showing the relationship of DFS (*P* = 0.400, n = 127) and OS (*P* = 0.183, n = 127) of CC patients and METTL3 expression in tumour cells. (C and D) Kaplan–Meier curves showing the relationship of DFS (*P* = 0.033, n = 127) and OS (*P* = 0.019, n = 127) of patients and METTL3 expression in TILs. (E and F) Kaplan–Meier curves showing the relationship of DFS (*P* = 0.393, n = 127) and OS (*P* = 0.227, n = 127) of CC patients and intratumoural CD33^+^ MDSC density. The percentages of DFS and OS were calculated by the Kaplan–Meier method, and *P* values were calculated by the log-rank statistic. Cut-off selection was based on X-tile.

## Data Availability

The authenticity of this article has been validated by uploading the key raw data to the Research Data Deposit (RDD) public platform (www. researchdata.org.cn).

## Abbreviations

METTL3: Methyltransferase-like 3
CC: Cervical cancer
MDSC: Myeloid-derived suppressor cells
DFS: Disease-free survival
OS: Overall survival
TNM: Tumour node metastasis
WHO: World Health Organization
T stage: Tumour status
N status: Lymph node metastasis
PBMC: Peripheral blood mononuclear cells
