# Supplementary figures and images for: Connecting METTL3 and intratumoural CD33+ MDSCs in predicting clinical outcome in cervical cancer
Source: J Transl Med. 2020 Oct 15;18:393. doi: 10.1186/s12967-020-02553-z (PMC7565373; doi:10.1186/s12967-020-02553-z)

Fig S1

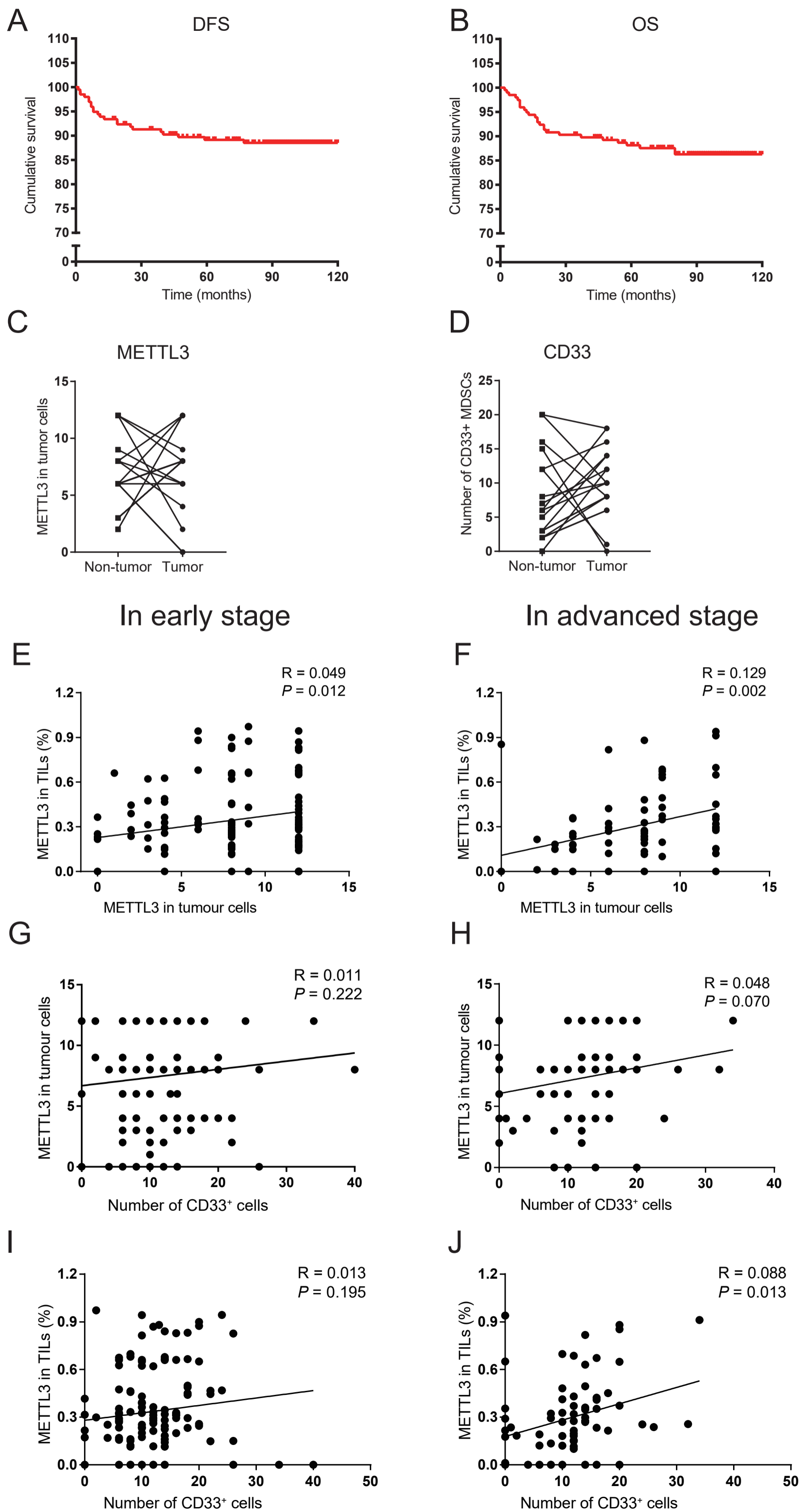

Supplement: Supplementary file 1 — Additional file 1: Figure S1. The DFS and OS curves of 197 CC patients, and expression of METTL3 and CD33 in tumours or in tumour-adjacent tissues. (A and B) Disease-free survival (DFS, A) and overall survival (OS, B) curves of 197 CC patients in this study. (C and D) Statistical analysis showing the comparison between the expression of METTL3 (P = 0.999, n = 18) and the number of CD33+ MDSCs (P = 0.295, n = 18) in tumour-adjacent tissues (Non-tumour) and tumour tissues (Tumour). (E and F) The association between METTL3 expression in tumour cells and the expression of METTL3 in TILs in early stage (E, R = 0.049, P = 0.012) or in advanced stage (F, R = 0.129, P = 0.002). (G and H) The association between METTL3 expression in tumour cells and intratumoural CD33+ MDSC number in early stage (G, R = 0.011, P = 0.222) or in advanced stage (H, R = 0.048, P = 0.070). (I and J) The association between METTL3 expression in TILs and intratumoural CD33+ MDSC number in early stage (I, R = 0.013, P = 0.195) or in advanced stage (J, R = 0.088, P = 0.013). Statistics were conducted with a paired Student’s t test in C and D. The correlation statistical analysis was performed using Spearman’s correlation and linear regression. R, Spearman’s correlation, is the correlation coefficient. [file 12967_2020_2553_MOESM1_ESM.pdf]

Fig S2 In tumour cells

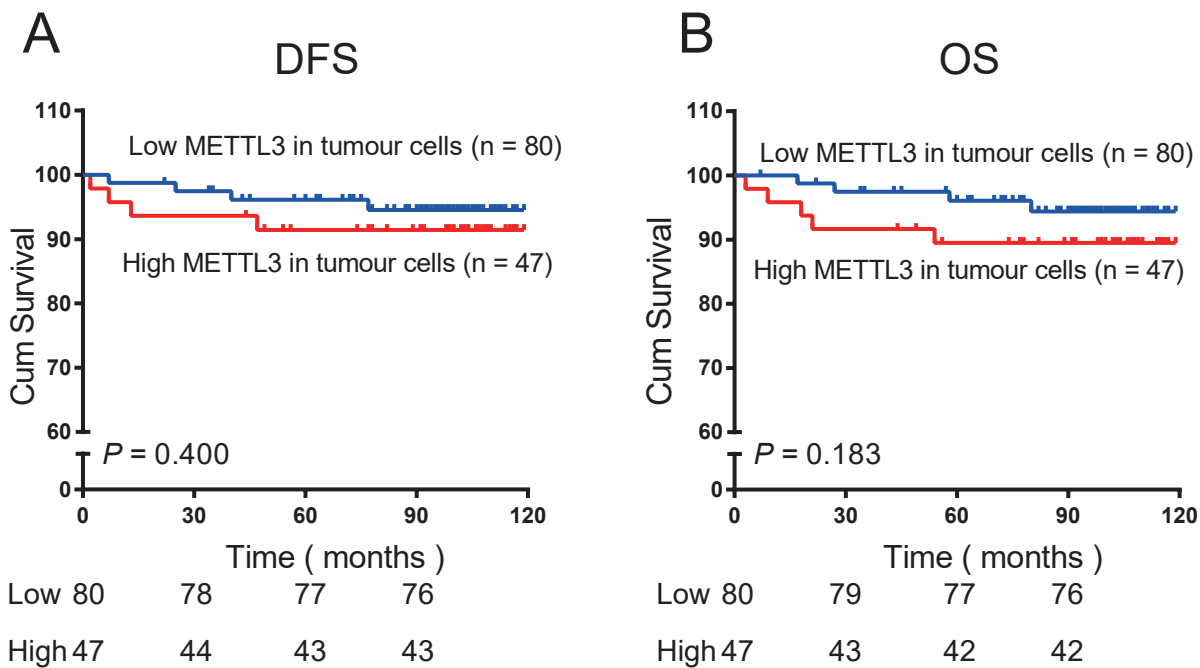

In TILs

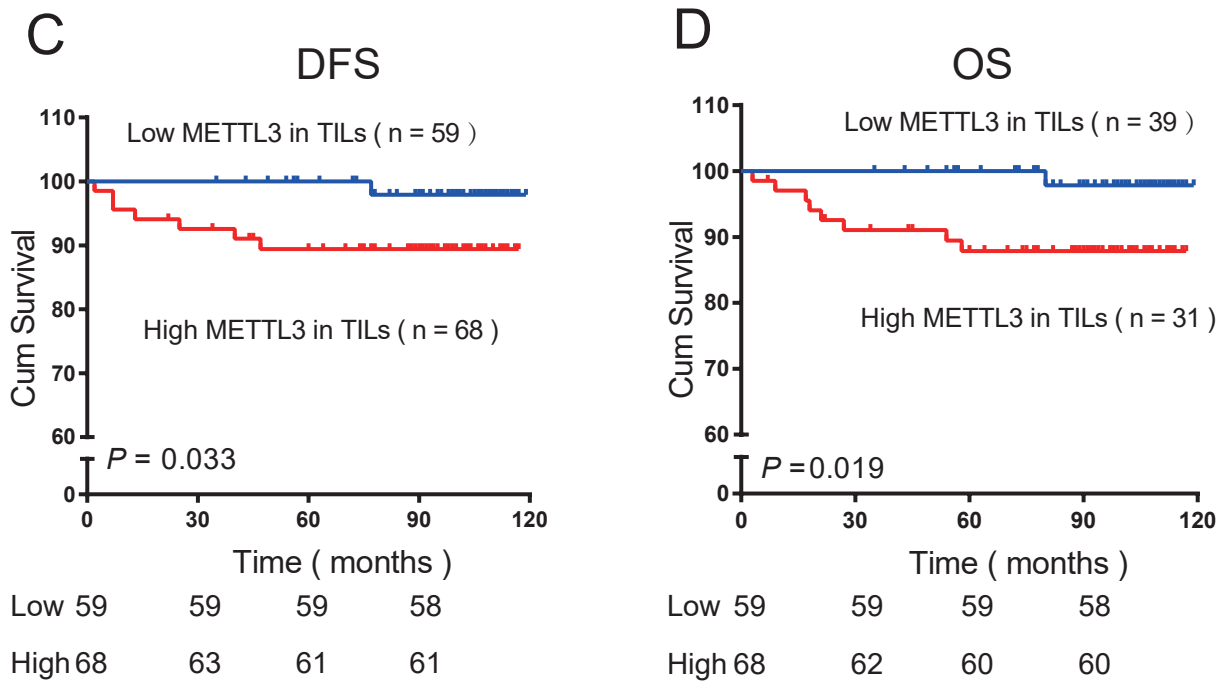

MDSCs

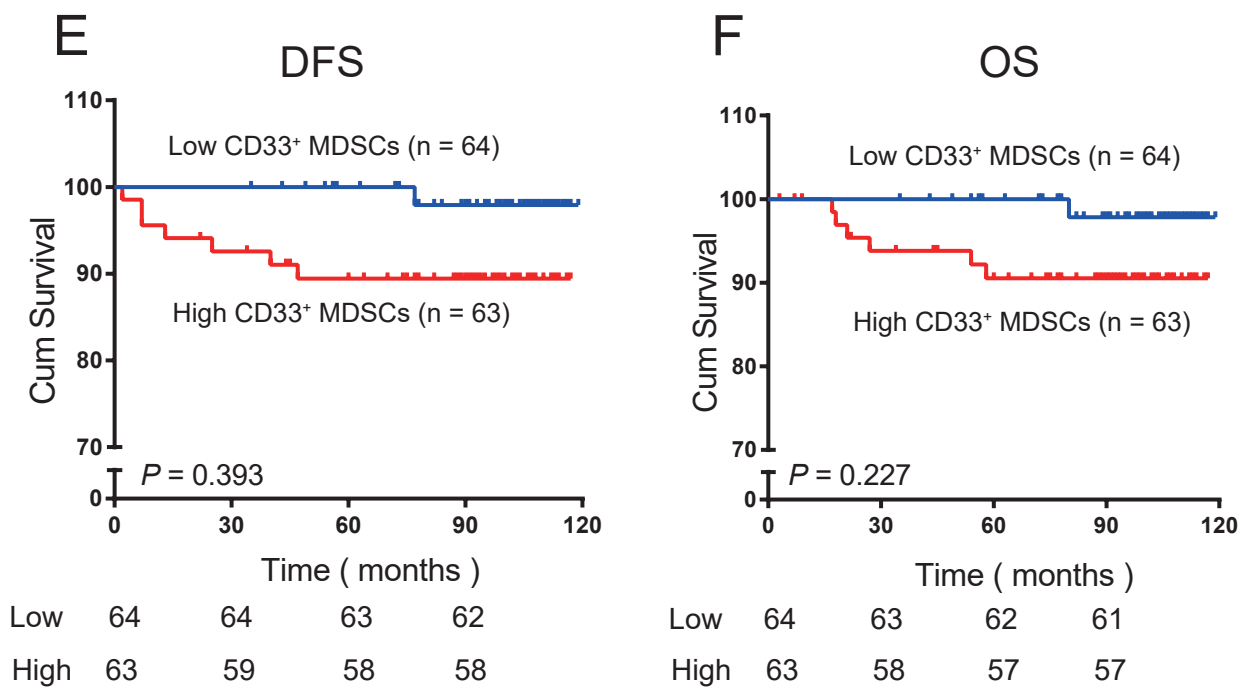

Supplement: Supplementary file 2 — Additional file 2: Figure S2. Kaplan–Meier curves of DFS and OS according to METTL3 expression in different cell populations and intratumoural CD33+ cells of early-stage patients. (A and B) Kaplan–Meier curves showing the relationship of DFS (P = 0.400, n = 127) and OS (P = 0.183, n = 127) of CC patients and METTL3 expression in tumour cells. (C and D) Kaplan–Meier curves showing the relationship of DFS (P = 0.033, n = 127) and OS (P = 0.019, n = 127) of patients and METTL3 expression in TILs. (E and F) Kaplan–Meier curves showing the relationship of DFS (P = 0.393, n = 127) and OS (P = 0.227, n = 127) of CC patients and intratumoural CD33+ MDSC density. The percentages of DFS and OS were calculated by the Kaplan–Meier method, and P values were calculated by the log-rank statistic. Cut-off selection was based on X-tile. [file 12967_2020_2553_MOESM2_ESM.pdf]
